# Burden of lymphoma in China, 1990−2019: an analysis of the global burden of diseases, injuries, and risk factors study 2019

**DOI:** 10.18632/aging.204006

**Published:** 2022-04-10

**Authors:** Weiping Liu, Jiangmei Liu, Yuqin Song, Xiaopei Wang, Lan Mi, Cai Cai, Donglu Zhao, Lijun Wang, Jun Ma, Jun Zhu

**Affiliations:** 1 Department of Lymphoma, Key Laboratory of Carcinogenesis and Translational Research Ministry of Education, Peking University Cancer Hospital and Institute, Beijing, China; 2 National Center for Chronic and Noncommunicable Disease Control and Prevention, Chinese Center for Disease Control and Prevention, Beijing, China; 3 Beijing Institute of Survey and Mapping, Beijing Municipal Key Laboratory of Urban Spatial Information Engineering, Beijing, China; 4 Department of Hematology and Oncology, Harbin Institute of Hematology and Oncology, Harbin, China

**Keywords:** lymphoma, Hodgkin disease, non-Hodgkin, incidence, mortality

## Abstract

Background: China is facing an aggravating disease burden of lymphoma. However, accurate information about lymphoma burden at the national and provincial levels is limited.

Results: The estimated number of disability-adjusted life years were 86,171.85 for Hodgkin lymphoma and 1,306,247.77 for non-Hodgkin lymphoma with the age-standardized rates of 4.95 and 71.00, respectively, per 100,000 population. There were estimated 9,468 new cases and 2,709 Hodgkin lymphoma-related deaths, and 91,954 new cases and 44,310 non-Hodgkin lymphoma-related deaths. Older individuals had a higher lymphoma burden. The age-standardized disability-adjusted life year rate in men was approximately two-folds higher than that in women. Moreover, disparities in lymphoma burden were observed across the provinces. Between 1990 and 2019, the disability-adjusted life year number decreased by 57.8% for Hodgkin lymphoma, and increased by 100.9% for non-Hodgkin lymphoma.

Conclusion: Burden of lymphoma showed heterogeneous change patterns varied according to sex, age, and provinces, with a steady decrease in Hodgkin lymphoma and a significant increase in non-Hodgkin lymphoma during the past three decades.

Methods: Following the analytical strategy used in the Global Burden of Diseases, Injuries, and Risk Factors Study 2019, age-, sex-, and province-specific incidence, mortality, and prevalence of Hodgkin lymphoma and non-Hodgkin lymphoma were analyzed. Lymphoma burden was assessed by incidence, mortality, prevalence, and disability-adjusted life year.

## INTRODUCTION

Hodgkin lymphoma (HL) and non-Hodgkin lymphoma (NHL) are common malignant lymphoid diseases that threaten public health. According to the GLOBOCAN 2020 statistics [1], the estimated worldwide number of incidence and deaths were 83,087 and 23,376 due to HL, and 544,352 and 259,793 due to NHL, respectively. Based on the Global Burden of Diseases, Injuries, and Risk Factors (GBD) Study 2019, the number and age-standardized rate of disability-adjusted life-years (DALYs) were 1,145,712 and 14.81 due to HL, and 6,991,329 and 90.36 due to NHL, respectively, whereas the age-standardized incidence and mortality rates per 100,000 population were 1.13 and 0.36 due to HL, and 5.91 and 3.29 due to NHL, respectively [2].

China has approximately one-fifth of the world’s population and is facing a dramatic disease burden of lymphoid neoplasms. The GBD 2019 showed that China accounted for 10.8% of new cases and 9.8% of deaths due to HL, and 20.1% of new cases and 17.4% of deaths due to NHL worldwide [2]. Based on data from the Chinese Center for Disease Control and Prevention’s disease surveillance points system, a study demonstrated an annual increase of 4.5% in the mortality rates of lymphoma and myeloma in China [3]. Moreover, there were significant sexual and geographical differences in the lymphoma burden in China [4, 5]. For example, the incidence of lymphoma in urban areas was 1.7 folds higher than that in rural areas (7.78 vs. 4.47, per 100,000 population), and the incidence of lymphoma in men was 1.3 folds higher than that in women (7.40 vs. 5.54, per 100,000 population) during the period of 1998-2010 in Beijing [6]. Therefore, comprehensive understanding of lymphoma burden will be helpful to develop disease control and prevention strategy.

A previous study based on the data of GBD 2016 demonstrated the burden decreased significantly for HL and increased for NHL in China from 2006 to 2016 [4]. However, updated epidemiologic information on lymphoma burden at the national and provincial levels is limited in China. On the other hand, the GBD 2019 presented updated estimates of disease burden due to 369 diseases and injuries in 204 countries from 1950 to 2019 [2]. To address this need, we conducted the present study, based on the data of GBD 2019, to provide epidemiological characteristics of lymphoma burden in China.

## RESULTS

### Lymphoma burden in China, 2019

The estimated DALY number and age-standardized DALY rate per 100,000 population were 86,172 [95% uncertainty interval (UI), 65,531–105,331] and 4.95 (95% UI, 3.85–6.08) due to HL, respectively; and 1,306,248 (95% UI, 1,103,334–1,521,347) and 71.00 (95% UI, 60.64–81.82) due to NHL, respectively. There were estimated 9,468 (95% UI, 7,082–11,481) new cases and 2,709 (95% UI, 2,001–3,293) HL-related deaths, and 91,954 (95% UI, 76,983–108,969) new cases and 44,310 (95% UI, 37,457–51,967) NHL-related deaths. The age-standardized incidence rates (ASIR) and age-standardized mortality rates (ASMR) per 100,000 population were 0.57 (95% UI, 0.43–0.69) and 0.15 (95% UI, 0.11–0.18) for HL, respectively; and 4.99 (95% UI, 4.24–5.87) and 2.32 (95% UI, 1.97–2.70) for NHL, respectively (Table 1). The estimated number of HL and NHL cases were 62,270 (95% UI, 46,300–375,760) and 410,380 (95% UI, 331,280–501,540), respectively, with the age-standardized prevalence rates per 100,000 population of 3.92 (95% UI, 2.96–4.75) for HL and 21.95 (95% UI, 17.94–26.65) for NHL.

**Table 1:** Age-standardized incidence, mortality, prevalence, YLLs, YLDs and DALYs rates in 2019.

**Variable**	**Hodgkin lymphoma**		**Non-Hodgkin lymphoma**	
	**Numbers (thousand)**	**Age-standardized rates (per 100,000)**	**Numbers (thousand)**	**Age-standardized rates (per 100,000)**
Incidence	9.47 (7.08−11.48)	0.57 (0.43−0.69)	91.95 (76.98−108.97)	4.99 (4.24−5.87)
Mortality	2.71 (2.00−3.29)	0.15 (0.11−0.18)	44.31 (37.46−51.97)	2.32 (1.97−2.70)
Prevalence	62.27 (46.30−375.76)	3.92 (2.96−4.75)	410.38 (331.28−501.54)	21.95 (17.94−26.65)
YLLs	80.71 (61.82−99.56)	4.62 (3.61−5.71)	1,246.19 (1,047.04−1,465.34)	67.79 (57.69−78.86)
YLDs	5.46 (3.49−7.69)	0.33 (0.21−0.46)	60.06 (41.57−81.57)	3.21 (2.23−4.34)
DALYs	86.17 (65.53−105.33)	4.95 (3.85−6.08)	1,306.25 (1,103.33−1,521.35)	71.00 (60.64−81.81)

Abbreviations: DALYs: disability-adjusted life years; YLDs: years lived with disability; YLLs: years of life lost. Data in parentheses are 95% uncertainty intervals.

### Lymphoma burden stratified by age and sex in 2019

**Figure 1:**
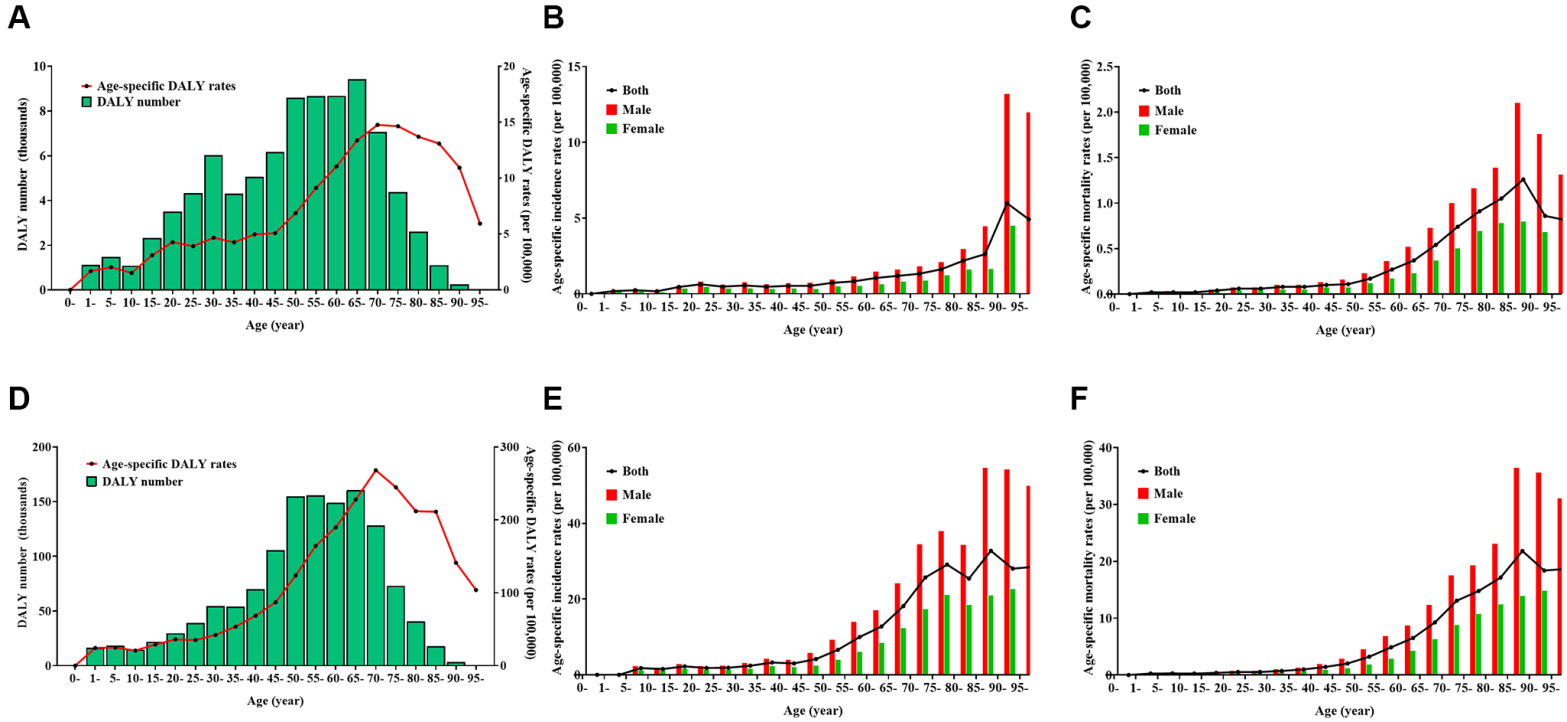
**Burden of Hodgkin lymphoma (HL) and non-Hodgkin lymphoma (NHL) by age and sex in 2019.** (**A**) number and age-standardized rates of disability-adjusted life years for HL, (**B**) age-standardized incidence rates for HL, (**C**) age-standardized mortality rates for HL, (**D**) number and age-standardized rates of disability-adjusted life years for NHL, (**E**) age-standardized incidence rates for NHL, (**F**) age-standardized mortality rates for NHL.

The age- and sex-specific lymphoma burdens are shown in Figure 1 and Supplementary Tables 1 and 2. In total, older individuals had a higher disease burden of both HL and NHL, in which men had a higher risk than women.

For HL, the DALY number was more than 5000 in the age groups 30–34 years and 40–74 years, and reached a peak in the age group of 65–69 years, whereas the age-specific DALY rate per 100,000 population was more than five in the age group of >45 years and reached a peak in the age group of 70–74 years (Figure 1A). The age-standardized DALY rate, ASIR, and ASMR per 100,000 population were 6.50 (95% UI, 4.61–8.58), 0.78 (95% UI, 0.50–0.98), and 0.20 (95% UI, 0.13–0.26), respectively, in men; and 3.44 (95% UI, 2.49–4.52), 0.40 (95% UI, 0.27–0.53), and 0.10 (95% UI, 0.07–0.13), respectively, in women. The age-specific incidence rates per 100,000 was more than one in the age group of >60 years, and reached a peak in the age group of 90–94 years in both sexes (Figure 1B). The age-specific mortality rates per 100,000 population was more than one in the age group of 80–89 years, and reached a peak in the age group of 85–89 years in both sexes (Figure 1C).

For NHL, the DALY number was more than 50,000 in the age group of 30–79 years and reached a peak in the age group of 65–69 years, whereas the age-specific DALY rate per 100,000 population was >50 in the age group of >35 years and reached a peak in the age group of 70–74 years (Figure 1D). The age-standardized DALY rate, ASIR, and ASMR per 100,000 population were 95.58 (95% UI, 77.16–116.59), 6.77 (95% UI, 5.44–8.35), and 3.19 (95% UI, 2.57–3.90) in men, respectively; and 46.95 (95% UI, 38.28–56.63), 3.36 (95% UI, 2.72–4.12), and 1.55 (95% UI, 1.25–1.88), respectively in women. The age-specific incidence rates per 100,000 population was more than 10 in the age group of >60 years, and reached a peak in the age group of 85–89 years in men and in the age group of >95 years in women (Figure 1E). The age-specific mortality rates per 100,000 population was more than 10 in the age group of >70 years, reaching a peak in the age group of 90–94 years in men and in the age group of >95 years in women (Figure 1F).

### Lymphoma burden stratified by provinces in 2019

**Figure 2:**
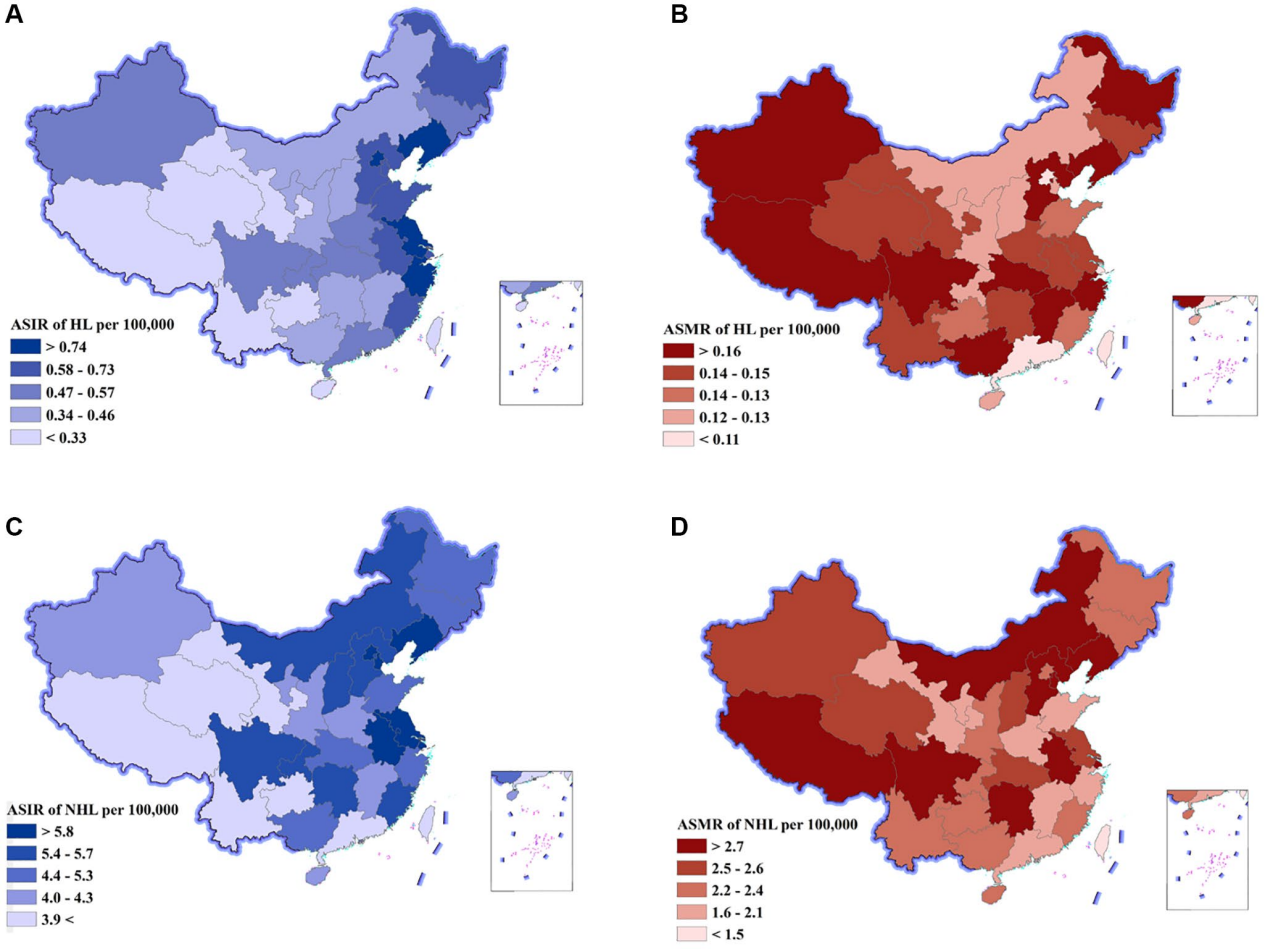
**Age-standardized incidence rates (ASIR) and age-standardized mortality rates (ASMR) of Hodgkin lymphoma (HL) and non-Hodgkin lymphoma (NHL) by province of China, 2019.** (**A**) ASIR of HL, (**B**) ASMR of HL, (**C**) ASIR of NHL, (**D**) ASMR of NHL.

The DALY number and age-standardized DALY rate at the province level are displayed in Table 2, Figure 2, and Supplementary Figure 1. In total, provinces with low sociodemographic index (SDI) often had a higher lymphoma burden, whereas provinces with high SDI had lower lymphoma burden. For HL, the DALY number was the highest in Hebei, Shandong, and Jiangsu, and the lowest in Macao special administrative region, Tibet, and Ningxia. The age-standardized DALY rate was the highest in Xinjiang, Hebei, and Tibet, and the lowest in Shanghai, Guangdong, and Beijing (Supplementary Figure 1A). The ASIR was the highest in Macao special administrative region, Hong Kong special administrative region, and Zhejiang, and the lowest in Guizhou, Tibet, and Qinghai (Figure 2A, Supplementary Figure 1B, Supplementary Table 3). The ASMR was the highest in Xinjiang, Hebei, and Tibet, and the lowest in Shanghai, Beijing, and Guangdong (Figure 2B, Supplementary Figure 1C, Supplementary Table 3).

**Table 2:** Number and age-standardized rate (ASR) of disability-adjusted life years of lymphoma by province of China in 2019 (per 100,000 population).

**Province**	**Hodgkin lymphoma**		**Non-Hodgkin lymphoma**	
	**Number**	**ASR**	**Number**	**ASR**
Anhui	3814.70 (4819.10−2704.16)	4.99 (6.24−3.62)	65327.02 (81757.05−51369.59)	79.70 (98.75−63.45)
Beijing	1208.69 (1559.84−830.47)	3.91 (4.95−2.76)	21703.19 (26417.95−17624.20)	69.26 (83.35−56.94)
Chongqing	1388.82 (2172.04−999.50)	4.16 (6.50−3.03)	26493.77 (33650.92−20584.39)	72.52 (91.77−56.75)
Fujian	2071.92 (2605.69−1570.45)	4.48 (5.61−3.41)	34617.94 (42542.65−28242.63)	71.08 (86.20−58.45)
Gansu	1380.95 (2299.69−943.66)	4.37 (7.38−3.00)	20612.83 (25280.02−16792.45)	61.62 (75.12−50.56)
Guangdong	5199.92 (6650.13−4062.56)	3.87 (4.94−3.06)	70097.51 (84980.76−57937.98)	51.65 (62.08−43.04)
Guangxi	2944.98 (4082.02−2219.44)	5.24 (7.19−3.98)	43259.33 (54220.78−33819.09)	73.67 (91.35−58.25)
Guizhou	1700.14 (3361.21−967.25)	4.22 (8.36−2.39)	30365.81 (39618.98−22900.06)	71.81 (93.34−54.58)
Hainan	429.51 (721.45−279.42)	3.92 (6.64−2.53)	7387.03 (9282.05−5815.52)	66.43 (82.81−52.81)
Hebei	6655.56 (9054.15−3685.41)	7.28 (9.81−4.14)	81673.89 (99629.40−64541.09)	84.97 (102.85−68.27)
Heilongjiang	2902.87 (3797.41−2038.33)	5.71 (7.42−4.16)	37269.14 (45959.34−29694.55)	68.03 (82.29−55.22)
Henan	5143.82 (7087.29−3913.16)	4.55 (6.24−3.49)	67927.77 (83753.21−53732.64)	57.42 (70.25−45.79)
Hubei	3659.36 (4742.45−2510.32)	5.05 (6.38−3.54)	57786.34 (73238.49−45533.42)	74.14 (92.44−59.27)
Hunan	3788.46 (5640.32−2775.22)	4.48 (6.66−3.31)	82080.24 (102211.78−65278.77)	91.14 (111.31−73.64)
Inner Mongolia	1310.33 (2106.82−920.46)	4.09 (6.61−2.92)	27461.06 (34125.39−21929.54)	80.33 (98.19−65.39)
Jiangsu	5584.82 (7675.73−3194.66)	5.09 (6.87−3.01)	85328.03 (107929.34−67519.57)	71.79 (89.38−57.27)
Jiangxi	2816.04 (3495.73−2119.85)	5.18 (6.43−3.92)	37412.82 (45815.01−30506.66)	66.38 (80.56−54.67)
Jilin	1762.10 (2235.43−1313.15)	4.83 (6.13−3.67)	29435.43 (37027.97−23943.62)	74.70 (92.85−62.11)
Liaoning	3813.69 (5067.05−2143.64)	6.18 (8.01−3.61)	59485.13 (75308.81−48160.19)	88.82 (108.57−72.62)
Ningxia	329.68 (537.09−229.21)	4.22 (6.86−2.95)	5033.67 (6382.65−3882.97)	63.27 (79.50−49.48)
Qinghai	331.80 (695.97−180.83)	4.76 (9.96−2.62)	5681.44 (7299.92−4365.89)	79.60 (100.20−62.49)
Shaanxi	2075.17 (3187.45−1458.56)	4.26 (6.60−3.01)	33897.25 (43143.69−25576.30)	66.38 (83.02−50.85)
Shandong	5834.93 (7410.87−4305.36)	4.56 (5.75−3.41)	79049.10 (97171.55−64109.46)	57.78 (70.26−47.31)
Shanghai	1316.75 (1720.12−909.00)	3.56 (4.51−2.53)	29457.48 (35950.71−23966.68)	78.30 (94.27−64.21)
Shanxi	1939.89 (2990.20−1360.85)	4.20 (6.45−2.97)	37104.63 (46868.15−28313.29)	76.49 (95.73−59.28)
Sichuan	5570.09 (7762.08−4152.04)	5.12 (7.11−3.90)	98315.43 (123040.54−78426.92)	84.04 (104.73−67.86)
Tianjin	832.72 (1044.54−616.02)	4.23 (5.25−3.26)	16772.06 (20125.30−13478.26)	82.34 (97.77−67.23)
Tibet	234.53 (483.13−129.60)	6.89 (14.21−3.86)	3060.66 (3941.10−2336.76)	90.66 (114.56−70.49)
Xinjiang	2281.96 (2998.27−1499.68)	8.70 (11.36−5.73)	20120.32 (25052.84−15974.66)	76.59 (93.68−61.53)
Yunnan	2694.83 (4696.48−1800.14)	4.88 (8.45−3.28)	39224.81 (49059.09−31772.38)	68.01 (83.68−55.41)
Zhejiang	4649.62 (6588.04−2315.18)	6.02 (8.50−3.06)	45069.51 (54668.56−36640.44)	55.38 (66.42−45.65)
Hong Kong^*^	457.71 (633.86−279.85)	4.13 (5.71−2.59)	7265.30 (9786.41−5389.27)	58.53 (78.31−43.33)
Macao^*^	45.49 (67.85−21.23)	5.00 (7.39−2.35)	471.82 (641.00−344.31)	49.40 (67.13−36.46)

^*^Special administrative regions. Data in parentheses are 95% uncertainty intervals.

For NHL, the DALY number was the highest in Sichuan, Jiangsu, and Hunan, and the lowest in Macao special administrative region, Tibet, and Ningxia. The age-standardized DALY rate was the highest in Hunan, Tibet, and Liaoning, and the lowest in Macao special administrative region, Guangdong and Zhejiang (Supplementary Figure 1D). The ASIR was the highest in Shanghai, Tianjin, and Liaoning, and the lowest in Tibet, Yunnan, and Gansu (Figure 2C, Supplementary Figure 1E, Supplementary Table 3). The ASMR was the highest in Liaoning, Hunan, and Sichuan, and the lowest in Macao special administrative region, Guangdong, and Shandong (Figure 2D, Supplementary Figure 1F, Supplementary Table 3).

### Trends in lymphoma burden from 1990 to 2019

**Figure 3:**
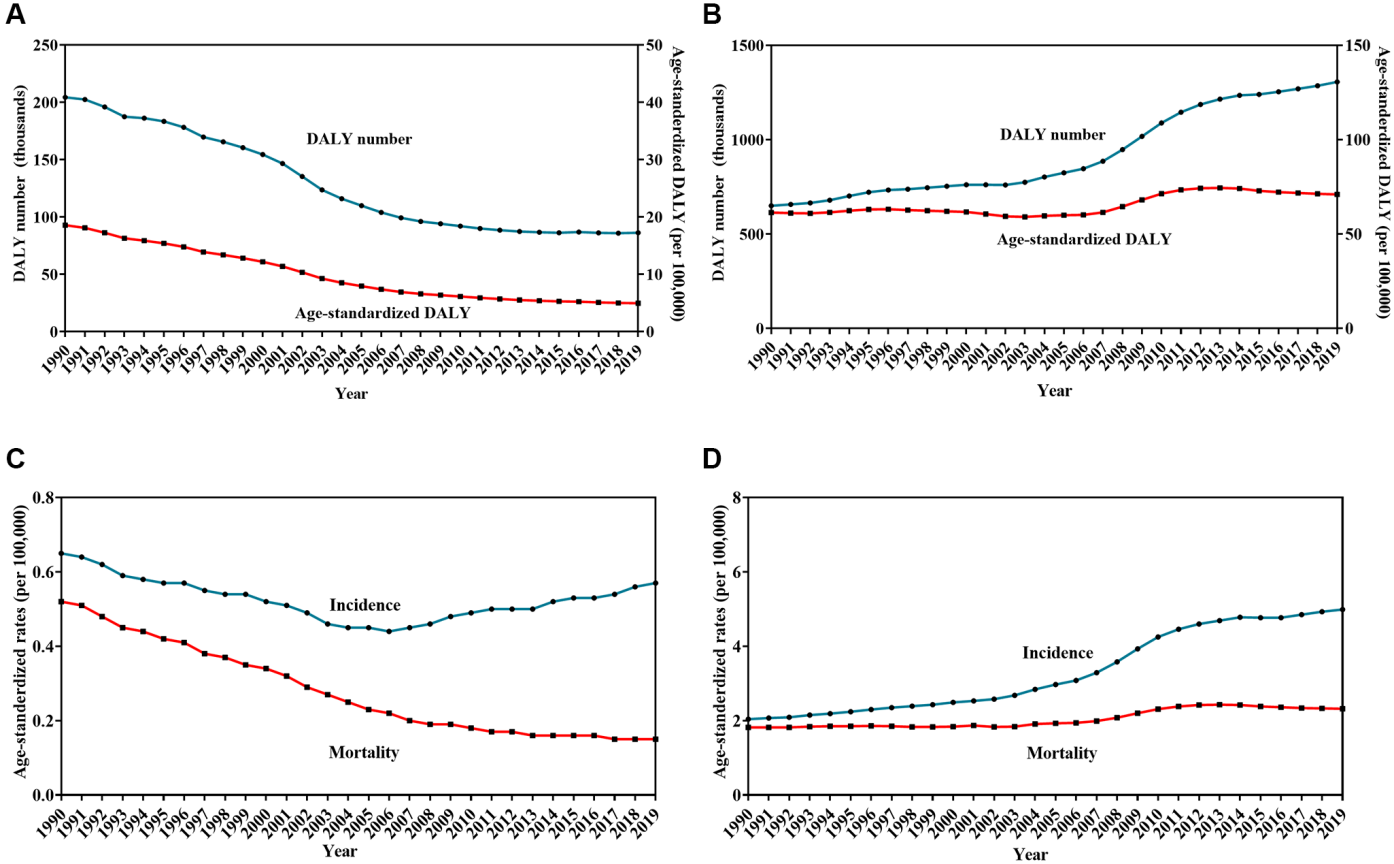
**Trends in burden of Hodgkin lymphoma (HL) and non-Hodgkin lymphoma (NHL) from 1990 to 2019.** Change in the number and age-standardized rates of disability-adjusted life years for HL (**A**) and NHL (**B**), Change in age-standardized incidence and mortality rates for HL (**C**) and NHL (**D**).

For HL, the DALY number and age-standardized DALY decreased by 57.8% and 74.4%, respectively, between 1990 and 2019 (Table 3, Figure 3A). The average annual percent change (AAPC) was −4.6 with 95% confidence interval (CI) of −4.8 to −4.4. The ASIR and ASMR showed a downward trend with a decrease of 12.3% (AAPC = −0.4, 95% CI: −0.6 to −0.2) and 71.2% (AAPC = −4.4, 95% CI: −4.5 to −4.2), respectively, over the past three decades (Figure 3C).

**Table 3:** Trends in lymphoma burden from 1990 to 2019.

**Variable**	**Trend 1**		**Trend 2**		**Trend 3**	
	**Year**	**APC (95% CI)**	**Year**	**APC (95% CI)**	**Year**	**APC (95% CI)**
**HL**						
Incidence	1990–2000	−2.09^*^ (−2.38, −1.81)	2000–2005	−3.36^*^ (−4.49, −2.22)	2005–2019	1.88^*^ (1.7, 2.05)
Mortality	1990–2000	−4.28^*^ (−4.49, −4.06)	2000–2008	−7.00^*^ (−7.36, −6.64)	2008–2019	−2.48^*^ (−2.67, −2.29)
Prevalence	1990–2005	2.57^*^ (2.33,2.81)	2005–2010	7.75^*^ (5.88, 9.66)	2010–2019	3.49^*^ (2.97, 4.02)
YLLs	1990–1999	−4.01^*^ (−4.38, −3.64)	1999–2008	−7.79^*^ (−8.21, −7.36)	2008–2019	−2.76^*^ (−3.04, −2.49)
YLDs	1990–2005	−0.32^*^ (−0.50, −0.14)	2005–2010	5.93^*^ (4.50, 7.37)	2010–2019	3.11^*^ (2.71, 3.51)
DALYs	1990–2000	−4.15^*^ (−4.48, −3.83)	2000–2007	−8.35^*^ (−9.02, −7.68)	2007–2019	−2.68^*^ (−2.93, −2.43)
**NHL**						
Incidence	1990–2004	2.16^*^ (2.02, 2.30)	2004–2011	7.51^*^ (6.96, 8.05)	2011–2019	1.28^*^ (0.95, 1.61)
Mortality	1990–2005	0.21^*^ (0.10, 0.32)	2005–2012	3.89^*^ (3.44, 4.34)	2012–2019	−0.86^*^ (−1.20, −0.52)
Prevalence	1999–2006	19.33^*^ (18.17, 20.50)	2006–2010	25.81^*^ (10.80, 42.85)	2010–2019	4.46 (2.06, 6.91)
YLLs	1990–2006	−0.37^*^ (−0.54, −0.20)	2006–2011	4.19^*^ (2.73, 5.67)	2011–2019	−0.67^*^ (−1.15, −0.18)
YLDs	1990–2003	4.13^*^ (3.90, 4.36)	2003–2011	10.70^*^ (10.07, 11.32)	2011–2019	2.70^*^ (2.23, 3.18)
DALYs	1990–2006	−0.28^*^ (−0.44, −0.11)	2006–2011	4.45^*^ (3.04, 5.88)	2011–2019	−0.55^*^ (−1.01, −0.08)

Abbreviations: APC: annual percentage change; CI: confidence interval; DALYs: disability−adjusted life years; YLDs: years lived with disability; YLLs: years of life lost. ^*^APC is significantly different from zero.

For NHL, the DALY number and the age-standardized DALY increased by 100.9% and 15.6%, respectively, between 1990 and 2019 (Table 3, Figure 3B). The AAPC was 0.4 with 95% CI of 0.2 to 0.7. The ASIR and ASMR per 100,000 population showed an upward trend with an increase of 14.2% (AAPC = 3.2, 95% CI: 3.0 to 3.3) and 21.9% (AAPC = 0.8, 95% CI: 0.7 to 1.0), respectively, over the past three decades (Figure 3D).

## DISCUSSION

Over the past three decades, lymphoma burden has manifested a heterogeneous change. The DALY number decreased by more than one-half due to HL and increased more than one-fold due to NHL. When removing the effects of population growth and aging, the disease burden of HL showed clear improvements, with age-standardized DALY decreasing by 74.4%, but the disease burden of NHL further deteriorating with age-standardized DALY rates increasing by 15.6%. This phenomenon can be partly attributed to population growth and aging. The National Bureau of Statistics of China reported that the national population increased from 1,143,330,000 in 1990 to 1,400,050,000 in 2019, whereas the population aged 65 years or older increased from 63,680,000 to 176,030,000 [7]. Moreover, China is moving from being an “aging society” to “aged society,” with an expected aging rate increasing from 10.92% in 2018 to 24.71% in 2044 [8]. Therefore, future studies are warranted to explore the correlation between lymphoma and changes in population size and structure.

The burden of lymphoma varied by age and sex. One study showed a bimodal distribution of global HL incidence with two peaks in the age groups of 20–39 years and >60 years, and a unimodal distribution of HL mortality with a significant increase in the age group of >50 years [9]. A study from Spain reported 2,403 deaths due to HL and 26,660 deaths due to NHL between 2008 and 2017, in which a peak occurred in the age group of 80–84 years in both sexes for HL, and in the age group of 75–79 in men and in 80–84 years in women for NHL [10]. In the United States of America, the incidence of diffuse large B-cell lymphoma and peripheral T-cell lymphomas reached a peak in the age group of 70–74 years, and it was approximately 50% higher in men than in women in 2016 [11]. In the present study, the DALY number of both HL and NHL reached a peak in the age group of 65–69 years and the age-specific DALY rate reached a peak in the age group of 70–74 years. Notably, male predominance of both HL and NHL was also seen in all age groups with about two folds sexual difference in the age-standardized DALY rate. These findings highlight the need to address the differential improvement of lymphoma burden based on age and sex differences, especially in the elderly population in China.

Another crucial factor influencing lymphoma burden is the improvement in access to health services [12]. To improve access to health services, the Chinese government has made substantial progress by enhancing financial protection and reforming the healthcare system [13]. For example, China established universal health insurance programs that covered 95% of urban and 97% of rural residents in 2011 [14]. Additionally, health resources and access in rural areas increased by approximately 50% in 2008–2014 [15]. Notably, the present study showed that the burden of NHL reached an inflection point with a conversion from increasing to decreasing in the age-standardized DALY rates and ASMR since 2011. Therefore, the findings of the current study support further research to explore the impact of accessibility and affordability of health services on the variation pattern of lymphoma burden.

With the establishment and promotion of clinical practice guidelines, the prognosis of patients with lymphoma has gradually improved [16]. A study from the National Central Cancer Registry of China showed that the 5-year overall survival (OS) of lymphoma improved gradually from 32.6% during 2003–2005 to 37.2% during 2013–2015 [17]. However, lymphoma was a group of heterogeneous diseases with different prognosis. For example, a study involving 3,760 patients with lymphoma from a tertiary hospital reported that the 5-year OS increased from 55.4% to 79.0% for classic HL, from 48.9% to 65.3% for mature B-cell lymphoma, and from 40.8% to 52.6% for peripheral T-cell lymphoma [18]. Therefore, it was needed to estimate the difference of disease burden among pathological subtypes in the future study.

Many factors such as socioeconomic status and health care access contribute to lymphoma burden [19–22]. The urban-rural discordance of mortality rates was partly due to poor availability of medical services and insufficient protection by healthcare insurance [3]. Those patients who lived in areas within the lower SDI quintiles often have poor survival due to insufficient access to timely diagnosis and treatment [17]. Additionally, increasing capacity for diagnosis, changes in cancer risk factors, and improvements in cancer registration contributed to the increasing age-standardized DALYs [23]. In the present study, disparities in lymphoma burden were observed across provinces. The age-standardized DALY rates of both HL and NHL showed a downward trend with the rise of SDI, which was high in provinces with low SDI, such as Sichuan and Hebei, and low in provinces with high SDI, such as Beijing and Shanghai. These findings underlined the correlation between imbalanced socioeconomic development status and geographical differences in lymphoma burden and suggest the urgency to apply the standardized procedures of diagnosis and treatment, especially in areas with low SDI.

This study has several limitations. First, it had all the limitations described in the GBD 2019. For example, the difference of disease burden among varied races or ethnicity was not analyzed because these data were not enrolled in the GBD database. Second, because the analyses were based on secondary estimated data rather than primary raw data, the impact of age and birth cohorts on the lymphoma burden was not evaluated. Third, except for SDI, other socio-demographic factors, such as urbanization and population migration [24], as well as environmental and metabolic factors [25], were not included in the analysis.

In conclusion, the present study provides a comprehensive spatiotemporal evaluation of lymphoma burden at the national and provincial levels in China. The disease burden of NHL has increased gradually, whereas the disease burden of HL has declined steadily over the last three decades. A huge distinction of lymphoma burden varying by age, sex, and region was also notable. The study findings will be useful for developing prevention and control strategies for lymphoma when a health policy is implemented in China.

## MATERIALS AND METHODS

We retrieved metadata of burden of lymphoma at the national and provincial level from the GBD 2019 database, which is available in the online GBD citation tool (http://ghdx.healthdata.org/gbd-2019). With permission of the Chinese Center for Disease Control and Prevention, the data at provincial level were available from the GBD 2019 database. The GBD 2019 methods, described previously [2, 26–28], was used to estimate the epidemiological quantity of lymphoma in China. International Classification of Diseases-10 (ICD-10) codes were used to represent HL (C81–C81.99) and NHL (C82–C86.6, C96–C96.9). Cause of death ensemble modelling (CODEm) was used to model mortality rates. Available data on causes of death were first standardized and then used to generate cause-specific mortality estimates and years of life lost. The sources of mortality data was based on the Disease Surveillance Points system and vital registration system from the Chinese Center for Disease Control and Prevention. Incidence and mortality data from cancer registries were processed to generate mortality-to-incidence ratios. During the process, the SDI, a composite indicator of background social and economic conditions influencing health outcomes in each location, was used as an important predictive covariate. The SDI was calculated as the geometric mean of total fertility rate for those younger than 25 years old, mean education for those 15 years old and older, and lag-distributed income per capita. Incidence estimates, generated by mortality to incidence ratios and mortality estimates, were combined with survival data to generate prevalence estimates and years lived with disability. DALYs were calculated by summing the years of life lost and years lived with disability. The DisMod-MR 2.1 tool was used to ensure consistency of estimation. To ensure consistency, we rescaled provincial level estimates to match GBD 2019 national level estimates as a whole.

The number and age-standardized rates of incidence, mortality, prevalence, and DALYs with 95% UI were used to represent burden of lymphoma. The age-standardized rates were calculated by direct method using the GBD world population. Temporal trends in burden of lymphoma from 1990 to 2019 were examined by fitting joinpoint models (version 4.6.0.0; National Cancer Institute). Temporal trends in burden of lymphoma from 1990 to 2019 were examined by joinpoint regression model (version 4.6.0.0; National Cancer Institute). The regression analysis started with logarithmic transformation of the rates. The grid search method and permutation testing were used to select the optimal joinpoint model. All models with a maximum of 2 joinpoints were applied in the present study. Furthermore, the annual percent change (APC) and AAPC with their corresponding 95% CI were determined for each segment of the joinpoint model and the entire study period, respectively. The term “decrease” or “increase” was used to describe the trends when the slope was statistically significant.

## Supplementary Materials

Supplementary Figure 1

Supplementary Tables
