# Supplementary material for: Burden of lymphoma in China, 1990−2019: an analysis of the global burden of diseases, injuries, and risk factors study 2019
Source: Aging (Albany NY). 2022 Apr 10;14(7):3175–90. doi: 10.18632/aging.204006 (PMC9037266; doi:10.18632/aging.204006)
Supplement: Supplementary Figure 1 [file aging-14-204006-s001.pdf]

SUPPLEMENTARY FIGURE

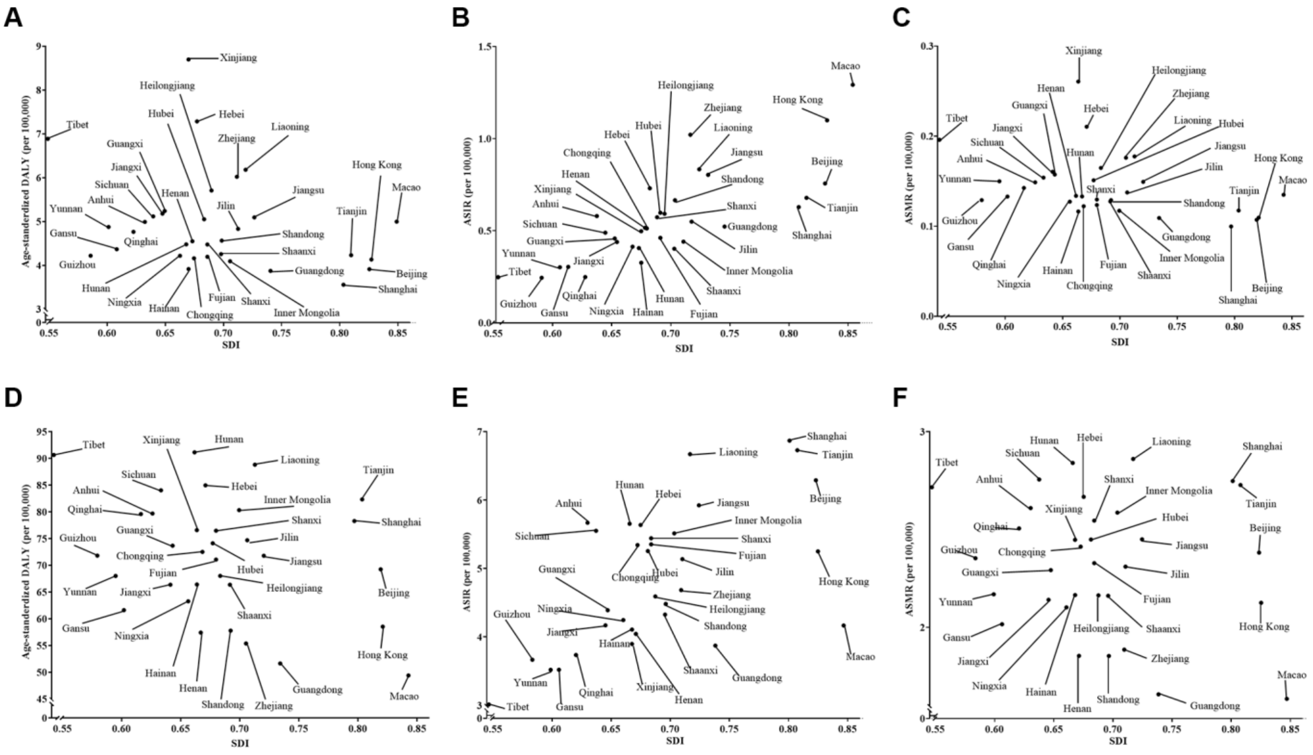

Supplementary Figure 1. Relation between lymphoma burden and socio-demographic index (SDI) at provincial level. (**A**) age-standardized disability-adjusted life years (DALYs) vs. SDI for Hodgkin lymphoma (HL), (**B**) age-standardized incidence rate (ASIR) vs. SDI for HL, (**C**) age-standardized mortality rate (ASMR) vs. SDI for HL, (**D**) age-standardized DALYs vs. SDI for non-Hodgkin lymphoma (NHL), (**E**) ASIR vs. SDI for NHL, (**F**) ASMR vs. SDI for NHL.
