# Supplementary material for: Burden of lymphoma in China, 1990−2019: an analysis of the global burden of diseases, injuries, and risk factors study 2019
Source: Aging (Albany NY). 2022 Apr 10;14(7):3175–90. doi: 10.18632/aging.204006 (PMC9037266; doi:10.18632/aging.204006)
Supplement: Supplementary Tables [file aging-14-204006-s002.pdf]

SUPPLEMENTARY TABLES

Supplementary Table 1. Age-specific incidence and mortality rates of Hodgkin lymphoma by age and sex in 2019 (per 100,000 population).

Age groups (year)	Incidence			Mortality		
	Both	Male	Female	Both	Male	Female
0–	0.00	0.00	0.00	0.00	0.00	0.00
1–	0.18 (0.14–0.27)	0.17 (0.11–0.29)	0.20 (0.14–0.32)	0.02 (0.01–0.03)	0.02 (0.01–0.03)	0.02 (0.02–0.03)
5–	0.24 (0.17–0.32)	0.33 (0.23–0.48)	0.13 (0.10–0.16)	0.02 (0.02–0.03)	0.03 (0.02–0.05)	0.01 (0.01–0.02)
10–	0.17 (0.13–0.23)	0.21 (0.15–0.32)	0.12 (0.09–0.18)	0.02 (0.02–0.03)	0.02 (0.02–0.04)	0.01 (0.01–0.02)
15–	0.46 (0.35–0.59)	0.54 (0.36–0.73)	0.37 (0.27–0.54)	0.04 (0.03–0.05)	0.05 (0.03–0.06)	0.03 (0.02–0.05)
20–	0.63 (0.48–0.84)	0.80 (0.52–1.07)	0.46 (0.31–0.69)	0.06 (0.05–0.08)	0.07 (0.05–0.10)	0.04 (0.03–0.07)
25–	0.48 (0.38–0.63)	0.59 (0.40–0.80)	0.37 (0.24–0.57)	0.06 (0.05–0.08)	0.07 (0.05–0.10)	0.05 (0.03–0.07)
30–	0.56 (0.42–0.71)	0.77 (0.49–1.01)	0.35 (0.24–0.53)	0.08 (0.06–0.10)	0.10 (0.07–0.14)	0.05 (0.04–0.07)
35–	0.47 (0.35–0.60)	0.63 (0.41–0.85)	0.31 (0.21–0.46)	0.08 (0.06–0.10)	0.10 (0.07–0.14)	0.05 (0.04–0.07)
40–	0.53 (0.38–0.67)	0.70 (0.46–0.95)	0.35 (0.23–0.49)	0.10 (0.08–0.13)	0.13 (0.09–0.18)	0.07 (0.05–0.09)
45–	0.53 (0.38–0.68)	0.73 (0.48–1.01)	0.31 (0.20–0.43)	0.11 (0.09–0.15)	0.16 (0.11–0.23)	0.07 (0.05–0.09)
50–	0.73 (0.52–0.92)	0.95 (0.60–1.30)	0.50 (0.33–0.69)	0.27 (0.20–0.34)	0.23 (0.15–0.31)	0.12 (0.08–0.16)
55–	0.83 (0.59–1.06)	1.14 (0.74–1.57)	0.52 (0.33–0.72)	0.37 (0.27–0.47)	0.36 (0.24–0.50)	0.17 (0.11–0.23)
60–	1.05 (0.73–1.33)	1.46 (0.90–1.95)	0.64 (0.38–0.87)	0.54 (0.39–0.67)	0.52 (0.36–0.70)	0.23 (0.15–0.31)
65–	1.19 (0.83–1.49)	1.61 (0.97–2.14)	0.79 (0.50–1.08)	0.74 (0.54–0.90)	0.73 (0.47–0.99)	0.37 (0.24–0.49)
70–	1.33 (0.91–1.63)	1.80 (1.09–2.37)	0.87 (0.53–1.16)	0.91 (0.70–1.23)	1.00 (0.64–1.32)	0.50 (0.32–0.66)
75–	1.63 (1.07–2.01)	2.10 (1.21–2.72)	1.21 (0.73–1.62)	0.91 (0.63–1.12)	1.16 (0.71–1.53)	0.69 (0.43–0.90)
80–	2.19 (1.38–2.72)	2.95 (1.63–3.90)	1.59 (0.94 –2.14)	1.05 (0.69–1.28)	1.39 (0.81–1.80)	0.78 (0.48–1.04)
85–	2.63 (1.63–3.29)	4.47 (2.39–5.82)	1.64 (0.92–2.20)	1.26 (0.82–1.52)	2.10 (1.21–2.69)	0.80 (0.48–1.07)
90–	5.99 (3.25–8.28)	13.21 (6.15–19.23)	4.49 (2.22–6.66)	0.86 (0.50–1.13)	1.76 (0.86–2.40)	0.68 (0.35–0.94)
95–	4.92 (2.38–7.44)	11.98 (5.25–20.89)	4.30 (1.91–6.68)	0.81 (0.41–1.18)	1.31 (0.60–1.93)	0.77 (0.37–1.14)

Data in parentheses are 95% uncertainty intervals.

Supplementary Table 2. Age-specific incidence and mortality rates of non-Hodgkin lymphoma by age and sex in 2019 (per 100,000 population).

Age groups (year)	Incidence			Mortality		
	Both	Male	Female	Both	Male	Female
0−	0.00	0.00	0.00	0.00	0.00	0.00
1−	0.00	0.00	0.00	0.28 (0.22−0.37)	0.31 (0.23−0.41)	0.26 (0.21−0.33)
5−	1.77 (1.44−2.21)	2.28 (1.78−2.95)	1.18 (0.98−1.45)	0.29 (0.24−0.36)	0.38 (0.30−0.48)	0.19 (0.16−0.23)
10−	1.53 (1.28−1.84)	1.81 (1.43−2.31)	1.21 (1.00−1.44)	0.26 (0.22−0.30)	0.31 (0.25−0.38)	0.20 (0.17−0.24)
15−	2.24 (1.85−2.70)	2.79 (2.14−3.60)	1.62 (1.36−1.95)	0.39 (0.32−0.46)	0.49 (0.37−0.61)	0.27 (0.23−0.32)
20−	1.81 (1.48−2.24)	2.31 (1.79−2.97)	1.27 (0.97−1.65)	0.53 (0.44−0.64)	0.68 (0.54−0.87)	0.35 (0.27−0.45)
25−	1.88 (1.56−2.29)	2.45 (1.97−3.06)	1.29 (0.94−1.71)	0.55 (0.47−0.65)	0.72 (0.59−0.88)	0.37 (0.28−0.48)
30−	2.40 (2.00−2.93)	3.16 (2.54−3.94)	1.63 (1.22−2.15)	0.72 (0.61−0.84)	0.96 (0.78−1.17)	0.48 (0.36−0.61)
35−	3.24 (2.70−3.95)	4.22 (3.32−5.36)	2.23 (1.67−2.89)	1.00 (0.84−1.18)	1.30 (1.04−1.61)	0.68 (0.51−0.86)
40−	3.01 (2.44−3.68)	3.99 (3.08−5.15)	1.99 (1.53−2.55)	1.43 (1.18−1.71)	1.92 (1.49−2.41)	0.93 (0.71−1.17)
45−	4.15 (3.30−5.16)	5.81 (4.35−7.70)	2.43 (1.85−3.12)	2.02 (1.64−2.47)	2.85 (2.15−3.69)	1.16 (0.90−1.46)
50−	6.59 (5.23−8.18)	9.24 (6.87−12.20)	3.91 (2.99−5.03)	3.22 (2.60−3.94)	4.55 (3.42−5.85)	1.88 (1.46−2.40)
55−	9.98 (7.93−12.35)	13.91 (10.39−18.30)	6.00 (4.63−7.67)	4.87 (3.93−5.96)	6.83 (5.17−8.75)	2.89 (2.26−3.67)
60−	12.76 (10.31−15.49)	17.06 (12.92−21.82)	8.43 (6.55−10.72)	6.51 (5.41−7.77)	8.73 (6.75−11.08)	4.27 (3.38−5.33)
65−	18.14 (14.89−22.02)	24.14 (18.64−30.52)	12.34 (9.81−15.65)	9.25 (7.75−10.86)	12.34 (9.76−15.39)	6.26 (5.00−7.69)
70−	25.70 (21.06−30.95)	34.43 (26.73−43.40)	17.39 (13.81−21.99)	13.08 (11.11−15.43)	17.55 (13.96−21.90)	8.82 (7.14−10.76)
75−	29.09 (24.21−34.64)	37.95 (30.31−46.80)	21.11 (16.65−26.37)	14.78 (12.61−17.21)	19.26 (15.61−23.61)	10.74 (8.71−13.06)
80−	25.42 (21.45−30.02)	34.37 (27.92−41.69)	18.42 (14.42−22.77)	17.13 (14.66−19.76)	23.09 (19.03−27.68)	12.46 (10.04−14.92)
85−	32.75 (27.51−38.41)	54.62 (45.78−63.72)	20.97 (16.21−26.45)	21.82 (18.70−24.78)	36.47 (31.13−42.12)	13.93 (10.86−16.98)
90−	28.04 (22.16−33.24)	54.21 (44.12−65.19)	22.60 (17.13−27.90)	18.39 (14.96−21.36)	35.61 (29.59−41.70)	14.81 (11.27−17.86)
95−	28.52 (21.26−35.73)	49.97 (40.55−59.70)	26.63 (19.08−34.22)	18.66 (14.07−22.44)	31.02 (25.12−36.64)	17.57 (12.71−21.54)

Data in parentheses are 95% uncertainty intervals.

Supplementary Table 3. Age-standardized incidence, mortality and prevalence rates of lymphoma by province of China in 2019 (per 100,000 population).

Province	Hodgkin lymphoma			Non-Hodgkin lymphoma		
	Incidence	Mortality	Prevalence	Incidence	Mortality	Prevalence
Anhui	0.58 (0.41−0.75)	0.15 (0.10−0.19)	3.95 (2.83−5.13)	5.67 (4.44−7.07)	2.61 (2.07−3.23)	26.55 (19.82−34.92)
Beijing	0.76 (0.52−0.97)	0.11 (0.07−0.14)	5.96 (4.14−7.68)	6.29 (5.04−7.62)	2.38 (1.93−2.88)	35.36 (26.87−44.82)
Chongqing	0.51 (0.37−0.80)	0.12 (0.09−0.19)	3.60 (2.54−5.62)	5.34 (4.15−6.81)	2.41 (1.89−3.04)	25.68 (18.89−34.30)
Fujian	0.60 (0.45−0.78)	0.13 (0.09−0.16)	4.30 (3.22−5.76)	5.35 (4.33−6.63)	2.33 (1.89−2.84)	26.37 (19.83−34.13)
Gansu	0.30 (0.21−0.50)	0.13 (0.09−0.22)	1.57 (1.04−2.70)	3.52 (2.87−4.36)	2.02 (1.65−2.43)	9.48 (6.50−13.03)
Guangdong	0.52 (0.39−0.68)	0.11 (0.08−0.14)	3.81 (2.85−4.99)	3.87 (3.12−4.77)	1.66 (1.37−2.00)	16.65 (12.06−22.04)
Guangxi	0.44 (0.33−0.61)	0.16 (0.12−0.21)	2.59 (1.91−3.61)	4.39 (3.44−5.44)	2.29 (1.78−2.87)	15.64 (10.88−21.22)
Guizhou	0.24 (0.14−0.49)	0.13 (0.07−0.25)	1.06 (0.58−2.22)	3.67 (2.76−4.81)	2.35 (1.80−3.06)	7.62 (4.52−11.14)
Hainan	0.33 (0.20−0.56)	0.12 (0.08−0.19)	1.94 (1.19−3.42)	4.11 (3.16−5.17)	2.16 (1.71−2.69)	14.50 (10.14−19.56)
Hebei	0.73 (0.41−0.99)	0.21 (0.12−0.29)	4.79 (2.69−6.63)	5.64 (4.54−6.98)	2.67 (2.14−3.23)	23.01 (16.85−30.72)
Heilongjiang	0.59 (0.43−0.78)	0.16 (0.11−0.21)	3.92 (2.87−5.31)	4.59 (3.69−5.69)	2.16 (1.77−2.63)	19.35 (14.20−25.59)
Henan	0.52 (0.39−0.72)	0.13 (0.10−0.18)	3.50 (2.61−5.00)	4.04 (3.28−5.05)	1.85 (1.48−2.28)	16.53 (11.57−22.26)
Hubei	0.57 (0.40−0.75)	0.15 (0.10−0.20)	3.86 (2.73−5.12)	5.26 (4.15−6.71)	2.45 (1.96−3.05)	23.79 (17.66−31.24)
Hunan	0.41 (0.30−0.62)	0.13 (0.10−0.20)	2.49 (1.81−3.85)	5.65 (4.61−7.00)	2.84 (2.27−3.49)	23.67 (17.79−30.94)
Inner Mongolia	0.44 (0.31−0.73)	0.12 (0.08−0.19)	2.98 (2.04−5.03)	5.51 (4.40−6.82)	2.59 (2.07−3.19)	24.63 (18.56−32.53)
Jiangsu	0.80 (0.48−1.10)	0.15 (0.08−0.21)	5.97 (3.62−8.16)	5.92 (4.59−7.55)	2.45 (1.92−3.07)	30.52 (22.58−39.83)
Jiangxi	0.46 (0.34−0.58)	0.16 (0.12−0.20)	2.71 (2.02−3.58)	4.17 (3.36−5.12)	2.14 (1.75−2.60)	15.77 (11.43−20.97)
Jilin	0.55 (0.41−0.71)	0.14 (0.10−0.17)	3.81 (2.80−5.04)	5.14 (4.26−6.35)	2.31 (1.98−2.81)	24.38 (18.72−32.03)
Liaoning	0.83 (0.49−1.10)	0.18 (0.10−0.24)	6.02 (3.60−7.91)	6.67 (5.37−8.27)	2.86 (2.32−3.53)	34.85 (26.59−45.51)
Ningxia	0.41 (0.29−0.66)	0.13 (0.09−0.20)	2.63 (1.80−4.37)	4.25 (3.34−5.27)	2.10 (1.64−2.62)	15.87 (11.49−21.08)
Qinghai	0.25 (0.14−0.51)	0.14 (0.08−0.29)	0.98 (0.51−2.09)	3.74 (2.94−4.68)	2.51 (1.98−3.08)	6.63 (4.08−9.84)
Shaanxi	0.40 (0.28−0.62)	0.13 (0.09−0.19)	2.53 (1.71−4.03)	4.33 (3.35−5.52)	2.16 (1.66−2.68)	17.11 (11.99−23.12)
Shandong	0.67 (0.48−0.85)	0.13 (0.09−0.16)	4.92 (3.54−6.32)	4.48 (3.57−5.55)	1.85 (1.50−2.26)	21.42 (15.37−27.65)
Shanghai	0.63 (0.45−0.84)	0.10 (0.07−0.13)	4.88 (3.54−6.55)	6.87 (5.49−8.40)	2.75 (2.21−3.33)	38.57 (29.69−49.20)
Shanxi	0.46 (0.32−0.72)	0.12 (0.09−0.19)	3.12 (2.11−4.99)	5.44 (4.19−6.74)	2.55 (1.96−3.13)	24.69 (18.03−32.43)

Sichuan	0.49 (0.36−0.67)	0.15 (0.12−0.21)	3.07 (2.24−4.36)	5.55 (4.38−6.95)	2.76 (2.22−3.43)	22.90 (16.77−30.97)
Tianjin	0.68 (0.49−0.88)	0.12 (0.08−0.15)	5.15 (3.81−6.75)	6.73 (5.39−8.30)	2.73 (2.18−3.29)	36.88 (27.86−46.98)
Tibet	0.25 (0.14−0.51)	0.20 (0.11−0.40)	0.53 (0.29−1.08)	3.01 (2.34−3.81)	2.72 (2.11−3.39)	2.12 (0.73−3.93)
Xinjiang	0.50 (0.32−0.66)	0.26 (0.17−0.34)	2.19 (1.37−3.00)	3.90 (3.20−4.86)	2.45 (2.00−3.02)	8.78 (5.72−12.55)
Yunnan	0.30 (0.20−0.52)	0.15 (0.10−0.25)	1.39 (0.89−2.46)	3.52 (2.85−4.33)	2.17 (1.78−2.65)	8.58 (5.68−11.99)
Zhejiang	1.02 (0.50−1.45)	0.18 (0.08−0.25)	7.71 (3.87−10.92)	4.68 (3.82−5.73)	1.89 (1.52−2.29)	23.17 (17.39−30.12)
Hong Kong*	1.10 (0.65−1.54)	0.11 (0.07−0.15)	8.83 (5.23−12.51)	5.25 (3.94−7.03)	2.13 (1.60−2.83)	27.69 (19.36−37.78)
Macao*	1.29 (0.59−1.94)	0.13 (0.06−0.20)	10.36 (4.76−15.60)	4.17 (3.09−5.60)	1.63 (1.21−2.18)	21.13 (14.69−29.02)

*Special administrative regions. Data in parentheses are 95% uncertainty intervals.
